#  Modelface: an Application Programming Interface (API) for Homology Modeling Studies Using Modeller Software

**Published:** 2016

**Authors:** Amirhossein Sakhteman, Bijan Zare

**Affiliations:** a *Department of Medicinal Chemistry, School of Pharmacy, Shiraz University of Medical Sciences, Shiraz, Iran. *; b *Medicinal and Natural Products Chemistry Research Center, Shiraz University of Medical Sciences Shiraz, Iran.*; c *School of Advanced Medical Sciences and Technologies, Shiraz University of Medical Sciences Shiraz, Iran.*

**Keywords:** Application Programming interface, Homology modeling, Batch scripting, Single point mutation, Missing atom types

## Abstract

An interactive application, Modelface, was presented for Modeller software based on windows platform. The application is able to run all steps of homology modeling including pdb to fasta generation, running clustal, model building and loop refinement. Other modules of modeler including energy calculation, energy minimization and the ability to make single point mutations in the PDB structures are also implemented inside Modelface. The API is a simple batch based application with no memory occupation and is free of charge for academic use. The application is also able to repair missing atom types in the PDB structures making it suitable for many molecular modeling studies such as docking and molecular dynamic simulation. Some successful instances of modeling studies using Modelface are also reported.

## Introduction

Homology modeling is a very common computational approach for representing the 3D structures of proteins when there is limitation in using experimental methods such as x-ray crystallography, NMR (nuclear magnetic resonance) or atomic force microscopy (1, 2). An application of comparative modeling is in microbial cell factories to solve problems on protein production (3). Other applications of homology modeling include designing site directed mutagenesis and studies related to protein-protein interaction (4). Furthermore, obtaining the 3D structure of the target protein is a vital step in structure based computational studies such as docking and molecular dynamic simulations (5). Different softwares have been developed by the time being to predict the 3D structures of proteins. MODWEB, IMPALA, PSI-BLAST, MODLOOP and MODELLER are some instances of the applications designed for comparative modeling studies (6, 7).

**Figure 1:**
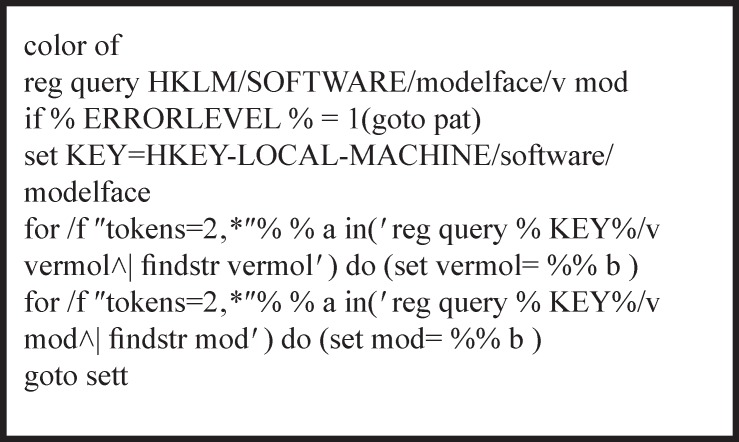
The codes relating to registry queries of Modelface

**Figure 2:**
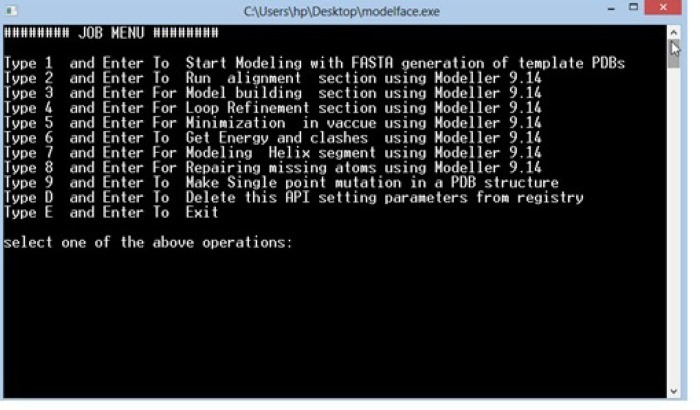
The Job tasks menu for Modelface

**Figure 3:**
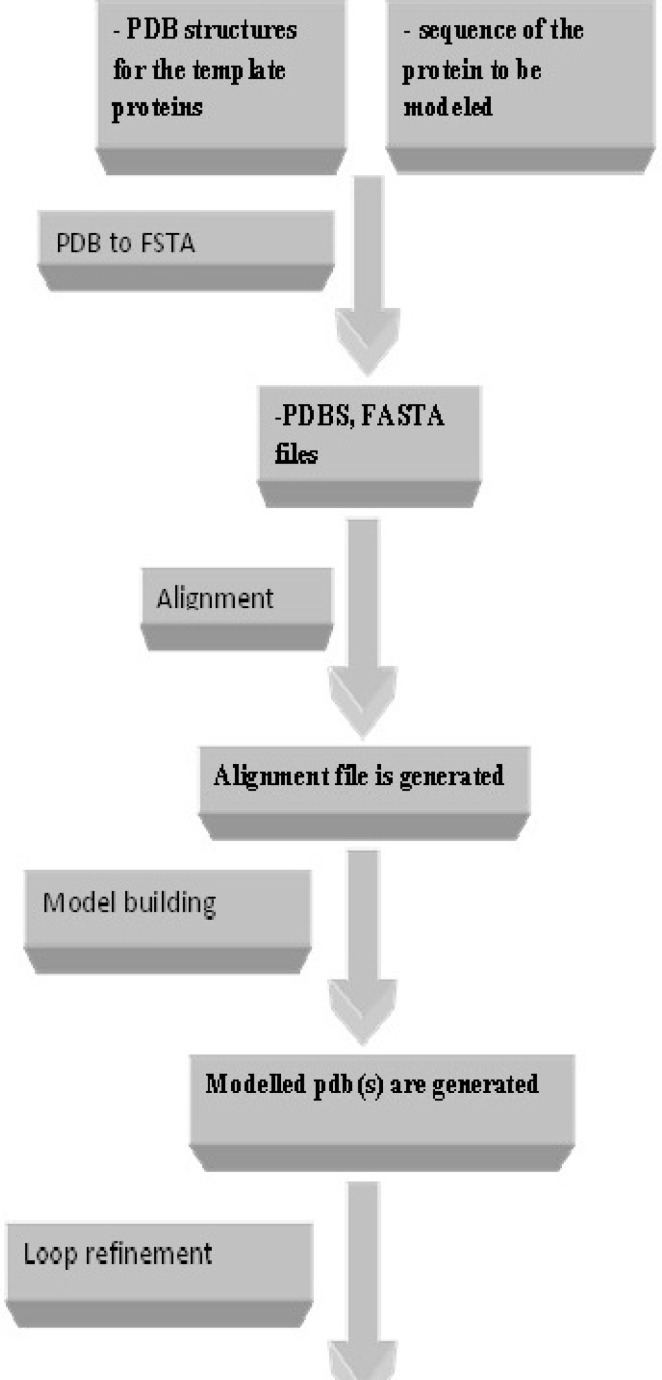
Successive steps of homology modeling using Modelface.

**Figure 4:**
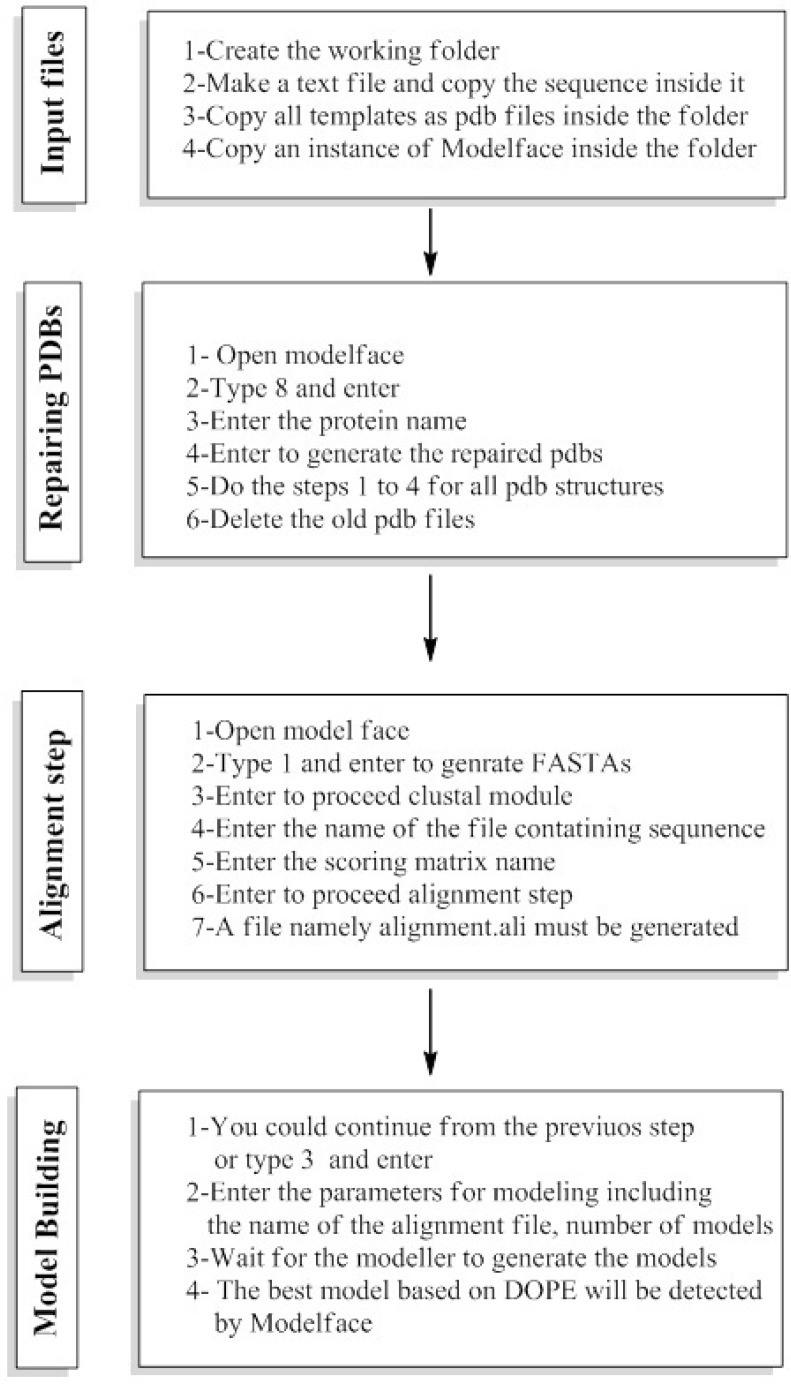
The experimental procedure of homology modeling using modelface

**Figure 5:**
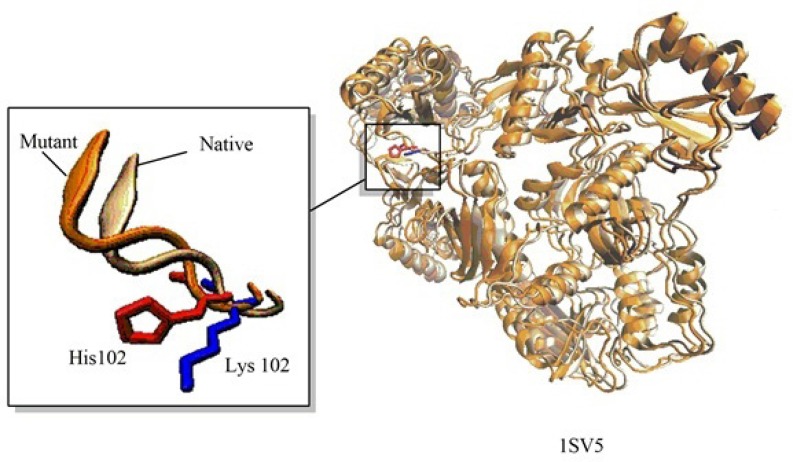
An example for Application of Modelface in making single point mutation in Human Immune deficiency (HIV-1) reverse transcriptase at Lys102. Visualization of the structures was performed using VMD(21)

Among the described applications, Modeller showed to be a successful package with different modules and abilities such as clustal, model building, loop refinement and energy calculation (8-10). Since Modeller is using python scripts for using the fortran libraries, it is performable on most platforms including windows, linux and Mac. However, a great deal with Modeller is that it is command based and no graphical interface is provided in the package. Therefore, the users need to be familiar with python scripting and the syntax of each command must be studied prior to generation of the proper python scripts (11). On the other hand python scripting is sensitive to some modifications such as indentations and the users must take special care in editing the python scripts (12). To cope with this problem many users developed different interfaces some of which are commercial or need other requirements such as Pymol or .Net framework for performance (11, 13-17). In an attempt to design a comprehensive toolbox with fewer requirements and more capabilities, an easy to use interface was prepared for windows by means of batch scripting. One advantage of this interface with respect to similar applications is that it does not need any requirements or libraries for performance. Furthermore, Modelface is working on both 32 and 64 bit systems and showed to be compatible on all windows platforms (Xp, vista, 7 and 8). Meanwhile, its ability to make single point mutations in PDB structures makes it suitable for many molecular modeling studies in the field of drug design and molecular modeling.

*Materials and methods*

All experiments were performed on a laptop running on windows 8. Windows notepad was used for writing the batch scripts using the standard CMD syntax for each command. Each module of the application was written separately in order to make debugging procedure more facile. A series of batch scripts were developed in the first section of the interface in order to check out Modeller executables. As displayed in Figure 1. this part is interfering with windows REGEDIT to make permanent registry query values in the operating system. The possibility for removing all added query values from the registry was implemented in Modelface in order to permit the users to update their Modeller versions or remove the API parameters. A job menu task was subsequently designed to perform the mostly used tasks of Modeller software. The correct formats of python scripts for different jobs including alignment, model building, loop refinement, helix generation, energy calculation and repairing missing atom types were thereafter implemented in the application. Optional parameters such as the ability to include or merge hydrogens, disulfide bridge, performing post molecular dynamics refinement are suggested to the user at each step. Different CMD loops were designed for reading pdb files and converting them into FASTA. This algorithm was based on the presence of Cα atoms in all residues. For this purpose, all PDB lines starting with Cα are being detected and the three letter codes corresponding to the residues are translated to one letter codes. The next piece of code makes it possible to consider all pdb files in the alignment file. Modelface was designed so that Model.py would be generated in another module of Modelface and the best models based on energy values are presented to the user. Some CMD commands such as findstr was used for reading key words in the resulted log files of Modeller(18). About 600 code lines were managed in this application. The guides for each step are displayed at each step to the user. To solve the problem of the files path, Modelface executable should be performed in the folder with templates and sequence. Modelface is therefore able to communicate with Modeller at any folders. The resulted batch script was finally compiled as a standalone executable (.exe) application by means of a bat to exe converter v1.6 (19). The final application was tested on other platforms including windows Xp, vista and 7.

Modelface is freely available for non-commercial use. It can be easily retrieved from http://salilab.org/modeller/wiki/Links. 

## Results and discussion

As displayed in Figure 2. different modules are provided in Modelface. The first four modules of Modelface are used for comparative homology modelling studies according to the procedure illustrated in Figure 3.

The first module of Modelface is a PDB to FASTA converter. This part of Modelface is responsible for translating three letter PDB coordinates of templates into one letter FASTA sequences. These FASTA sequences are needed for running the alignment section. Modelface is designed to cope with both single and multiple alignment modelling strategies. Therefore, in cases that only one PDB is present in the working directory, single alignment files will be generated. On the other hand, multiple alignment files will be generated if more template PDBs are present in the working directory. A successful instance of running Modelface for G-protein coupled receptors is reported for human dopamine D4 receptor based on four template PDBs (PDB codes = 4GRV, 3UON, 3PBL and 4IB4) (5). The third module of Modelface is responsible for building the final models. The best model of the protein will be presented at the end of the procedure based on DOPE scores. Some features of Modeller, neglected in other softwares, such as symmetry of the resulted proteins are provided in Modelface. Taking care of symmetry is important in homology modelling of the channels. Meanwhile the conserved disulfide bridge as well as MD optimization could be applied in the modelling procedure. In cases that certain loop regions of the receptor should be subjected to refinement section, as implemented in Modeller software, the fourth module of Modelface can easily be utilized. A feature of Modelface is that all needed parameters for running Modeller including the path to Modeller executables are saved in registry of Windows operating system. This feature permits the user to communicate with Modeller without running the Modeller from its shell. As seen, most Modelface modules are batch scripts for generation of Modeller python files. Another feature of Modelface is its ability to repair missing atom types of PDB structures. Most protein structures are resolved by X-ray crystallography and in many cases missing atom types are present in the PDB files. Missing atom types could lead to some problems in homology modelling, molecular dynamic and docking simulation studies. For this purpose, pre-processing of PDB structures before any in silico studies is highly recommended. This module of Modelface can be also used for removing the co-crystal ligands and water molecules prior to docking simulation studies. De novo modelling of helix structures is a module of Modelface which can be used in certain circumstances. The procedure of homology modelling with known templates is displayed in Figure 4.

These steps include preparation of the input files, repairing the missing atom types, alignment section and model building. The explicit procedure together with the trial movies are provided in supplementary information of the manuscript. A unique feature of Modelface which is not seen in other application programming interfaces of Modeller is its ability to make single point mutations in PDB structures. During this procedure the native PDB structure of the protein is used as template for generation of mutants. As an example, a mutation of human immune deficiency virus 1 (HIV-1) reverse transcriptase at residue number 102 was carried out by Modelface. As seen in Figure 5. Lys102 was replaced by His102 in the mutant form. This feature of Modelface could be used in many in silico studies such as docking simulations (20). In addition, MD simulation in the gas phase, finding clashes of nearby atoms and bonding energy calculations are other features of Modelface application.

The most important features of Modelface can be summarized as bellow:

- No dependencies to any other applications or libraries rather than Modeller9.x
- Executable on all windows platforms (xp, vista, 7 and 8) and compatible with 32 and 64 bit systems
- A pdb to fasta converter is implemented to get ease of using other softwares during homology modeling procedure (Figure 2.)
- Compatible on Modeller 9.10 and higher versions of Modeller
- Ability to run clustal, model building and loop refinement steps, successively (Figure 1.)
- Supporting most used modules of Modeller including energy and clashes calculation, repairing missing atom types, *de novo* helix generator, structure minimization and homology modeling
- Taking the parameters in each step, interactively
- Ability to generate and run python scripts according to the user’s input.
- Notifying the users before running Modeller allowing the advanced users to modify the python scripts, if needed
- Step by step help before running each module
- All Modeller environments and path options are implemented in the application so that there is no need to open the Modeller shell separately.
- Possibility to make single point mutations in the PDB structures

The advantages of the software include easy handling of python scripts for modeller, facile way of making single point mutation in PDB structures and an interactive wizard shape environment for step by step performing of modeling experiments. This application will be supported based on the future releases of modeller and the feedback of the users. Meanwhile, the source code for the batch scripts are provided in supplementary information. A limitation of this application which will be considered in the future versions is its DOS shape environment. 

This limitation could be resolved by compiling a visual .NET application with ability to make batch scripts.

## Conclusion

An API was presented in this study in order to use different modules of Modeller software in an interactive view. The interface was tested in different windows platforms and its applicability have been verified. Due to presence of some useful features including single point mutations, repairing missing atom types and de novo helix generation, the interface can be considered as a notable bioinformatics toolbox in studies related to drug design and molecular modeling. In addition, the interactive feature of the interface and its stepwise mechanism permits the facile use of Modeller modeling features for almost all users. 
